# Differentiating Intracellular from Extracellular Alkaline Phosphatase Activity in Soil by Sonication

**DOI:** 10.1371/journal.pone.0058691

**Published:** 2013-03-07

**Authors:** Shuping Qin, Chunsheng Hu, Oene Oenema

**Affiliations:** 1 Key Laboratory of Agricultural Water Resources, Center for Agricultural Resources Research, Institute of Genetic and Developmental Biology, The Chinese Academy of Sciences, Shijiazhuang, Hebei, China; 2 Wageningen University and Research, Alterra, Wageningen, The Netherlands; Wageningen University, The Netherlands

## Abstract

Differentiating intracellular from extracellular enzyme activity is important in soil enzymology, but not easy. Here, we report on an adjusted sonication method for the separation of intracellular from extracellular phosphatase activity in soil. Under optimal sonication conditions [soil:water ratio  =  1/8 (w/v) and power density  =  15 watt ml^-1^], the activity of alkaline phosphomonoesterase (phosphatase) in a Haplic Cambisol soil increased with sonication time in two distinct steps. A first plateau of enzyme activity was reached between 60 and 100 s, and a second higher plateau after 300 s. We also found that sonication for 100 s under optimal conditions activated most (about 80%) of the alkaline phosphatase that was added to an autoclaved soil, while total bacteria number was not affected. Sonication for 300 s reduced the total bacteria number by three orders of magnitude but had no further effects on enzyme activity. Our results indicate that the first plateau of alkaline phosphatase activity was derived from extracellular enzymes attached to soil particles, and the second plateau to the combination of extracellular and intracellular enzymes after cell lysis. We conclude that our adjusted sonication method may be an alternative to the currently used physiological and chloroform-fumigation methods for differentiating intracellular from extracellular phosphatase activity in soil. Further testing is needed to find out whether this holds for other soil types.

## Introduction

Phosphomonoesterase (phosphatase) activities are widely investigated in soils since they catalyze the hydrolysis of ester–phosphate bonds, releasing the inorganic phosphate, which can be assimilated by plants and micro-organisms [1]–[3]. The overall phosphatase activities in soils are composed of extracellular activity from accumulated phosphatase adhered to soil particles and intracellular phosphatase in living microorganisms [4]. As yet, it is still a challenge to discriminate intracellular enzyme activity from extracellular enzyme activity in soil [5]–[10]. Among the numerous attempts to address this issue, the physiological method [11] and the chloroform-fumigation method [9] were most widely used. A critical assumption in the physiological method is that the results from the enzyme assays are a combination of intracellular and extracellular activity [11]. However, a direct correlation between the enzyme activity and the size of the microbial biomass is sometimes absent [12]. The chloroform-fumigation method assumes that the enzymatic activity measured before chloroform-fumigation originates from the extracellular enzyme activity, while that after chloroform-fumigation from the combination of extracellular and intracellular activity [9]. However, the released intracellular enzyme might be partially degraded by protease following chloroform-fumigation [13]. A rapid fumigation method (5 min) was proposed to minimize proteolysis [10].

We explored the potentials of sonication to discriminate between intracellular versus extracellular phosphatase activity in soil. Sonication is a wave generated by vibration [14]. It generally causes both heating and cavitation in liquid surroundings [15]. Sonication is widely used for the determination of particle-size distribution [16] and fractionation of organic matter [17]. Short-time (30 s) sonication with low-power intensity was reported to not significantly decrease the total coliform number of wastewater particles until the power exceeded 30 watt (W) [18]. In contrast, long-time sonication (150 s) was reported to be able to lyse soil microbial cells and release the intracellular compounds [19]. The above results suggest that sonication might be a promising tool for differentiating between extracellular and intracellular enzyme activity in soil. Earlier, De Cesare et al. [20] explored the potential of sonication for evaluating the activity of immobilized acid phosphatase. Acid phosphatase activity was increased in a clay loam soil after 120 s sonication, due to both of breakdown of soil aggregates and detachment of extracellular enzyme from soil particle surface, rather than the release of intracellular enzyme from proliferating cells. In this study, we further investigated the potential of sonication for discriminating extracellular versus intracellular alkaline phosphatase activity in a Haplic Cambisol.

We hypothesized that short-time sonication would activate the extracellular enzymes bonded with soil particles, while long-time sonication would lyse the microbial cell and release the intracellular enzymes.

## Materials and Methods

### Soil collection and processing

Soil samples were collected from the Luancheng Agro-ecosystem Station (37^°^90'N, 114^°^67'E, elevation 50 m) from the Chinese Academy of Sciences. The soil is classified as a silt loam Haplic Cambisol (FAO classification system; 13.8% sand, 66.3% silt, and 19.9% clay, pH 8.2, bulk density 1.44 g cm^-3^, organic carbon 12.05 mg g^-1^ and total nitrogen 1.12 mg g^-1^). The cropping system is winter wheat (mid-October to early-June) with summer corn (early-June to late September). After removing the crop residues, five soil cores (4.8 cm×10 cm depth) were randomly sampled and thoroughly mixed, ground through a 2-mm sieve and stored at 4^o^C. Before each experiment, soils were pre-incubated at 25°C for five days to activate microbial activity following the cold storage.

### Sonicator testing

Aliquots of 100 ml deionized water were sonicated for 100 s at power densities of 0, 5, 10, 15, 20 W ml^-1^, following the procedure of De Cesare et al. [20]. The increase of water temperature with the sonication time was monitored by a temperature sensor in the sonicator (JYD-650, 20 kHz, ZhiSun Instrument Co., Shanghai, China). The effects of the sonicator on temperature were analyzed statistically via regression analysis.

### Ultrasonic effects on soil dispersion

This experiment was carried out to find the optimal soil-water ratio (w/v). Different ratios (1:4, 1:6, 1:8, 1:10, w/v) were subjected to sonication at different power densities (0, 5, 10, 15, 20, 25, 30 W ml^-1^). Moist soil samples equivalent to 2.50, 1.66, 1.11 and 1.00 g dry weight and portions of 10 ml 50 mM borax-borate buffer (pH 8.2) were mixed in 20 ml cylindrical glass jars each. The jars were put in an ice bath to prevent heating during sonication. After 10 s gentle stirring and 180 s equilibration, the mixtures were sonicated (probe at 15 mm depth, 2 s burst and 2 s rest) for 300 s at power density of 0, 5, 15, 20, 25, 30 W ml^-1^, respectively. If the temperature of soil suspension exceeded 15°C, the temperature sensor will stop the sonicator automatically until the temperature decreased to below 13°C. The sonicated samples were equilibrated at 37^o^C for 10 min under gentle agitation. Then the suspensions were centrifuged at 12,000 *g* for 2 min. The absorbance of the supernatants were measured by a spectrophotometer at 410 nm.

### Ultrasonic effects on alkaline phosphatase and total bacterial number

Moist soil samples (1.11 g, dry weight) were mixed with 10 ml 50 mM borax-borate buffer (pH 8.2) in 20 ml cylindrical glass vessels and sonicated (probed at 15 mm depth) at power density of 10, 15, and 20 W ml^-1^ for 0, 20, 60, 80, 100, 150, 200, 250, 300, 350, 400 and 500 s, respectively.

Alkaline phosphatase activity was spectrophotometrically determined at 410 nm using *p*-Nitrophenyl-phosphate (40 mM) as substrates, according to the procedure of De Cesare et al. [20], with slight modifications. Instead of 0.5 M acetate buffer (pH 5) we used 50 mM borax-borate buffer (pH 8.2). Two controls were included: one was to remove the absorbance of chromophores that were released during the sonication, the other was to remove the *p*-Nitrophenyl-phosphate-hydrolyzing activity of non-enzymatic components in soil.

Total bacteria numbers (Log_10_ CFU g^-1^ dry soil) of the sonicated and the non-sonicated soil were determined using the most probable number (MPN) methods with beef extract–peptone substrate [21].

### Ultrasonic effects on commercial alkaline phosphatase activity

In order to simulate the ultrasonic effects on extracellular alkaline phosphatase activity, 30 µl 50 mU alkaline phosphatase from *Escherichia coli* (Sigma No. P-4252, St. Louis, USA) was added into 10 ml 50 mM borax-borate buffer (pH 8.2). Autoclaved (121 ^o^C, 1 h) soil samples (1.11 g, dry weight), which had no alkaline phosphatase activity, were added into the buffer. Then the slurry was shaken for 30 s, equilibrated for 15 min at 25°C, and sonicated at a power density of 15 W ml^-1^ for 0, 20, 60, 80, 100, 150, 200, 250, 300, 350, 400 and 500 s, respectively. Control samples (without autoclaved soil) were identically and synchronously treated. The sonication procedure and determination of alkaline phosphatase activity (mU ml^–1^) were the same as described above.

### Statistics

Differences between means were analyzed statically using the software SPSS 13.0 (SPSS Inc, 2004), and were compared by the LSD test at *P*<0.05

## Results

### Temperature change by sonication

**Figure 1:**
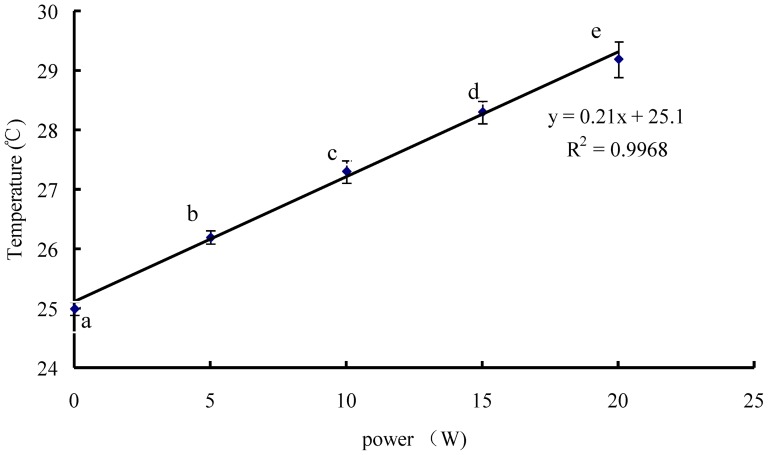
Temperature of 100 ml deionized water as a function of sonication power.

The water temperature increased nearly linearly with the power density of the sonicator (*P* < 0.05) (Fig. 1); for each power unit (W) increase, temperature increased 0.21 °C after sonication for 100 s. Increasing sonication time increased temperature also linearly (not shown).

### Soil dispersion by sonication

**Figure 2:**
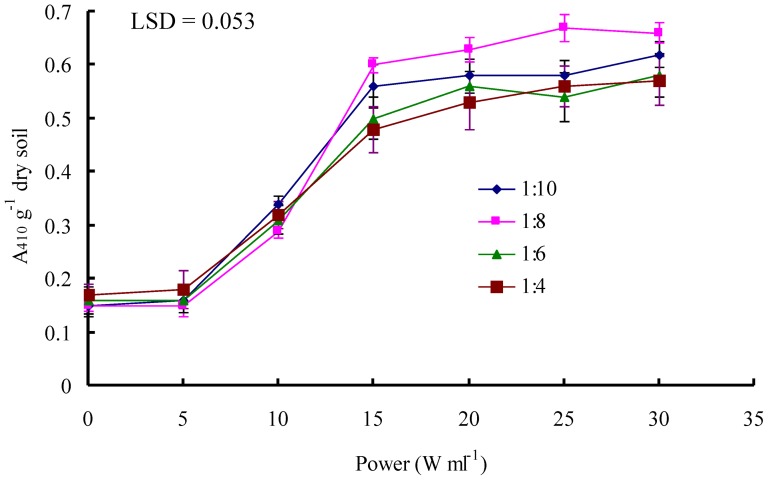
Absorbance (410 nm) of supernatants of soil slurries after 300 s sonication at different extraction ratios as a function of power density.

The absorbance of soil supernatants (after centrifugation at 12,000 *g* for 2 min) increased significantly when the ultrasonic power density increased from 5 to 15 W ml^-1^ (Fig. 2). A further increase of the ultrasonic power density from 15 to 30 W ml^-1^ did not contribute much to a further increase. The highest absorbance was found for a soil-water ratio of 1:8 (w:v), but differences between soil-water ratios were relatively small, though statistically significant (Fig. 2).

### Changes in phosphatase activity by sonication

**Figure 3:**
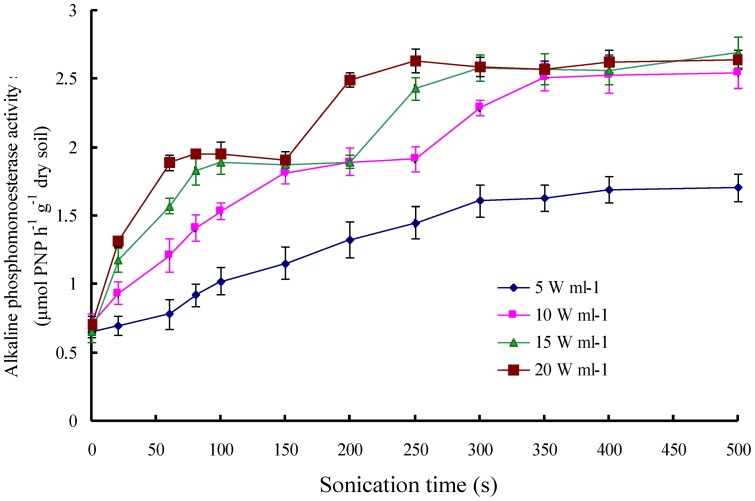
Activity of alkaline phosphatase of the field-moist soil as a function of sonication time. Data are means ± SD (*n*  =  3).

At relatively low power density (5 W ml^-1^), alkaline phosphatase activity steadily increased with sonication time until a plateau was reached at 1.8 to 1.9 µmol *p*-nitrophenol h^-1^ g^-1^ dry soil (Figure 3). Increasing the power density from 5 to 10, 15 and 20 W ml^-1^ decreased the time needed to reach the plateau at 1.8 to 1.9 µmol *p*-nitrophenol h^-1^ g^-1^ dry soil. Continued sonication further increased phosphatase activity at high power density, until a second plateau was reached at about 2.5 µmol *p*-nitrophenol h^-1^ g^-1^ dry soil (Figure 3). This step-wise increase of the phosphatase activity with sonication time is in line with our hypothesis and suggest that the activity related to the first plateau is from extracellular phosphatase and that the activity related to the second plateau is from extracellular plus intracellular phosphatase.

### Changes in extracellular phosphatase activity by sonication

**Figure 4:**
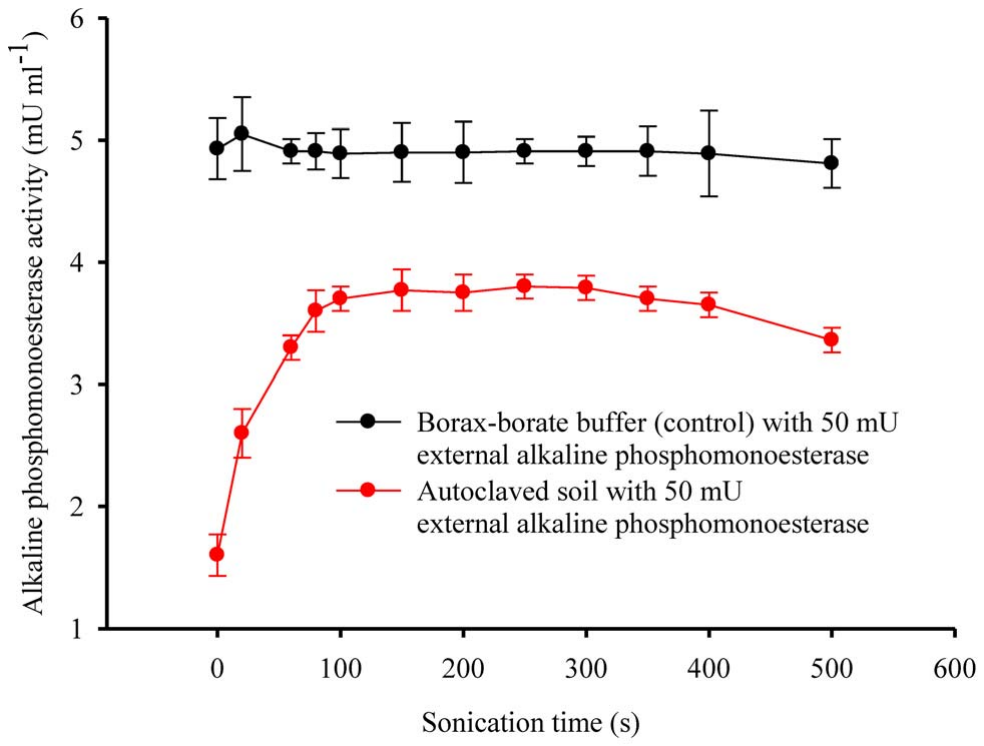
Activity of alkaline phosphatase of the autoclaved soil and the borax-borate buffer (control) with 50 mU external alkaline phosphatase as functions of sonication time.

The activity of added extracellular phosphatase to an autoclaved borax-borate buffer (control treatment) was quite stable against sonication (Fig. 4). In contrast, the activity of added extracellular phosphatase to autoclaved soil increased significantly with sonication time, until a plateau was reached (Fig. 4). Hence, sonication activated the extracellular external phosphatase from soil particles.

**Figure 5:**
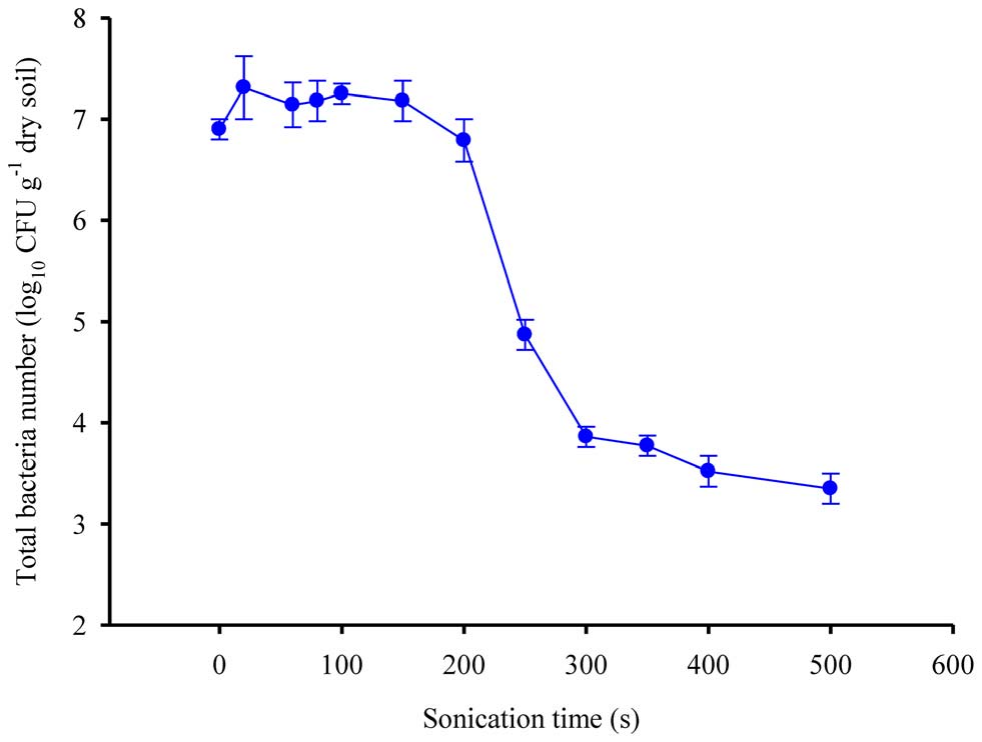
Total bacteria number of field-moist soil as a function of sonication time.

The total counts of bacteria (Log CFU g^-1^ dry soil) remained rather constant with an increase in sonication time until about 150 s. Thereafter, bacteria counts decreased, especially between 200 and 300 s. Total counts of bacteria decreased by three orders of magnitude when sonication time increased to 500 s (Fig. 5).

## Discussion

### Ultrasonic effects on soil dispersion

The increase in heat absorbed by the water was linearly related to the energy output of the sonicator under all power densities (Fig. 1), indicating the reliability of the sonicator applied. Also, sonication time and energy could be easily adjusted, and our temperature-regulated sonication prevented excessive heating of the samples.

Some spectrophotometrically active chromophores may be released from soil colloids during sonication treatment [20], and the increase in the absorbance of chromophores indirectly reflects the degree of soil dispersion [20]. Our results showed that the soil:water ratio was not very critical in the range of 1:4 to 1:10 (w:v) (Figure 2). Because of the highest absorbance at a soil:water ratio of 1:8 (Figure 2), we adopted this ratio in the subsequent experiments.

### Ultrasonic effects on phosphatase activity

The soil native alkaline phosphatase activity increased in two steps with an increase in sonication time, when sonication energy was in the range of 10–20 W ml^-1^ (Figure 3). This result contrasts with the results obtained with De Cesare et al. [20], who observed only one plateau of acid phosphatase activity. At low energy sonication, i.e. 5 W ml^-1^, we observed only one plateau (the lower one), and at high energy sonication (20 W ml^-1^) we observed that the first and second plateaus were reached soon (Figure 3).

Two independent mechanisms have been proposed to explain the ultrasonic effects on alkaline phosphatase activity. The first relates to the activation of the immobilized extracellular phosphatase in soil particles following its exposure [22], and the second to the release of intracellular phosphatase after cell lysis [20]. Short-time sonication (less than 200 s) appears effective for extracting living bacteria from soil [18], [23], [24]. In this study, 200 s sonication at a power density of 15 W ml^-1^ did not significantly decrease bacterial counts (Fig. 5), indicating that 200 s sonication at the power density of 15 W ml^-1^ did not cause cell lysis and very likely did not release intracellular enzymes. The first plateau of phosphatase activity in this study was thus probably related to the activation of extracellular enzyme in soil aggregates. Independent proof for this hypothesis was obtained from the experiment in which extracellular phosphatase was added to autoclaved soil. About 80 % of the added phosphatase was activated after 100 to 200 s sonication at a power density of 15 W ml^-1^ (Fig. 4).

Alkaline phosphatase can be excreted by both plant roots and soil microbes [25]-[26]. Microbes, especially bacteria, are able to produce and release large amounts of extracellular phosphatase due to their large biomass, high metabolic rate and short lifecycles [27]. The total number of bacteria in this study decreased by three orders of magnitude at high-energy sonication (4,500 J ml^-1^) for 150 to 500 s (Fig. 5). Stevenson [23] also reported that 300 s sonication significantly decreased the number of soil bacteria at a soil:water ratio of 1∶10 (both soil type and energy not described). Vargas et al. [28] reported that 240 s sonication with an amplitude of 20 µm caused cell disruption and release of invertase from a fungi (*Aspergillus niger*) in a liquid culture. Those results clearly suggest that sonication at high energy-level may destroy soil microbial cells and thereby may release intracellular alkaline phosphatase. Therefore, the second plateau of phosphatase activity observed in this study after sonication at relatively high-energy sonication of long duration was very likely related to a combination of extracellular phosphatase plus intracellular phosphatase released after microbial cell lysis.

### Potential of sonication to differentiate extracellular from intracellular phosphatase

Our results showed that low-energy sonication was able to activate the majority of adscititious (simulated extracellular) alkaline phosphatase but did not decrease the bacteria number. In contrast, high-energy sonication significantly decreased the number of bacteria (Fig. 5). These results indicate that extracellular alkaline phosphatase may be discriminated from intracellular alkaline phosphatase by differentiating the energy-level and duration of sonication. In this study, the extracellular activity could be discriminated from the intracellular alkaline phosphatase activity by step-wise sonication for 500 s at energy levels of 10 to 20 W ml^-1^ (Figure 3). It is worthy to note that the sonication effects on soil bacterial cell lysis was not comprehensively considered since the MPN method only determine 1-10% of soil micro-organisms.

Sonication is a promising alternative for differentiating extracellular from intracellular phosphatase activity in soils. It is worthy to note that the extracellular alkaline phosphatase activity was two times higher after sonication for 300 s than before sonication (Fig. 3 and 4). This was probably due to the activation of entrapped phosphatase in soil aggregates [22]. The activation of entrapped phosphatase was considered a disadvantage of sonication for assaying extracellular enzyme activities [8]. However, soil enzyme activity reflects the potential rather than the actual *in situ* activity [29]–[31].

## Conclusions

A sonication pretreatment offers the potential to quantify two levels of phosphatase activity, i.e., (i) potential extracellular activity, measured after high-energy sonication for 100-150 s., and (ii) potential extracellular plus intracellular activity, measured after high-energy sonication for >300 s. Applied level of energy and the duration of the sonication as well as the time-resolution of the enzyme activity measurements are critical. Further experimentations are needed to test whether sonication is also suitable for discriminating intracellular from extracellular enzyme activities in other soils.
